# The Emerging Roles of the RNA Binding Protein QKI in Cardiovascular Development and Function

**DOI:** 10.3389/fcell.2021.668659

**Published:** 2021-06-16

**Authors:** Xinyun Chen, Jianwen Yin, Dayan Cao, Deyong Xiao, Zhongjun Zhou, Ying Liu, Weinian Shou

**Affiliations:** ^1^Department of Pediatrics, Wells Center for Pediatric Research, Indiana University School of Medicine, Indianapolis, IN, United States; ^2^Guangdong Key Laboratory for Genome Stability and Human Disease Prevention, Department of Biochemistry and Molecular Biology, School of Basic Medical Sciences, Shenzhen University, Shenzhen, China; ^3^Department of Foot, Ankle and Hand Surgery, Shenzhen Second People’s Hospital, First Affiliated Hospital of Shenzhen University, Shenzhen, China; ^4^Faculty of Medicine, School of Biomedical Sciences, The University of Hong Kong, Hong Kong

**Keywords:** QKI, pre-mRNA processing, cardiovascular system, development, function

## Abstract

RNA binding proteins (RBPs) have a broad biological and physiological function and are critical in regulating pre-mRNA posttranscriptional processing, intracellular migration, and mRNA stability. QKI, also known as Quaking, is a member of the signal transduction and activation of RNA (STAR) family, which also belongs to the heterogeneous nuclear ribonucleoprotein K- (hnRNP K-) homology domain protein family. There are three major alternatively spliced isoforms, QKI-5, QKI-6, and QKI-7, differing in carboxy-terminal domains. They share a common RNA binding property, but each isoform can regulate pre-mRNA splicing, transportation or stability differently in a unique cell type-specific manner. Previously, QKI has been known for its important role in contributing to neurological disorders. A series of recent work has further demonstrated that QKI has important roles in much broader biological systems, such as cardiovascular development, monocyte to macrophage differentiation, bone metabolism, and cancer progression. In this mini-review, we will focus on discussing the emerging roles of QKI in regulating cardiac and vascular development and function and its potential link to cardiovascular pathophysiology.

## Introduction

Transcriptional and posttranscriptional modifications are critical in regulating gene expression (Day and Tuite, 1998). Posttranscriptional Pre-mRNA splicing removes intron sequences from newly transcribed immature and unstable pre-mRNAs, which is essential for generating mature and protein-coding mRNAs (Lee and Rio, 2015). In general, splicing events are either constitutive splicing or alternative splicing. Constitutive splicing is considered a ubiquitous pre-mRNA processing event; however, a recent investigation demonstrated that the constitutive splicing efficiency increases with the transcription rate, which is a phenomenon called “economy of scale splicing.” This finding suggests that constitutive splicing has a unique function in amplifying transcription (Ding and Elowitz, 2019). In comparison to constitutive splicing, alternative splicing is a tightly regulated event that is controlled upon unique cellular differentiation states and distinct physiological states, and it results in multiple mRNA isoforms from a single gene by specific inclusion or exclusion of certain exons (Zhu et al., 2017). Ninety-five percent of the human genome has been estimated to undergo some level of alternative splicing (Wang et al., 2008), and these events are closely associated with normal developmental processes and physiological functions as well as various congenital malformations and pathophysiological conditions.

The regulation of alternative splicing is composed of various *cis-*regulatory nucleotide sequences and *trans-*acting factors (Schaal and Maniatis, 1999; Fairbrother et al., 2002; Wang et al., 2004; Wang and Wang, 2014). RNA-binding proteins (RBPs) can recognize specific RNA sequences to form ribonucleoprotein complexes, which regulates diverse biological functions in pre-mRNA processing, such as pre-mRNA splicing, RNA modification, and RNA transportation (van den Hoogenhof et al., 2016). Many RBPs is known to play particularly critical roles in pre-mRNA splicing (Blech-Hermoni and Ladd, 2013; Ladd, 2016). In response to the upstream biological signals, these RBPs coordinate alternative splicing events by binding to unique nucleotide sequences within intron regions and specific exons (e.g., splicing enhancers or silencers) to either promote or inhibit specific alternative splicing events. The serine/arginine-rich (SR) protein family and the heterogeneous nuclear ribonucleoprotein (hnRNP) protein family are the most well-known RBPs (Zahler et al., 1992; Martinez-Contreras et al., 2007; Long and Caceres, 2009).

## QKI Plays an Important Role in Pre-mRNA Processing

**FIGURE 1:**
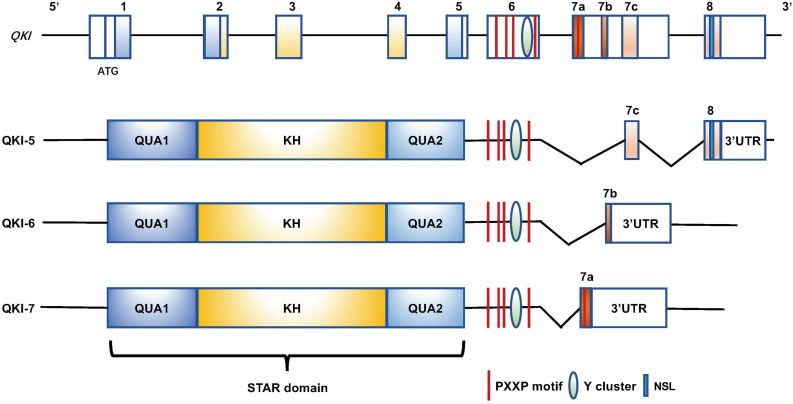
Genomic and protein domain structure of the human *QKI* gene and QKI isoforms (QKI-5, QKI-6, and QKI-7). All isoforms contain an RNA-binding motif (KH domain) that is flanked by QUA1 and QUA2 domains. The proline-rich PXXP motifs and the tyrosine cluster are at the C-terminus, and they exhibit distinct C-terminal structures resulting from alternative splicing of QKI primary transcripts. QKI-5 has a nuclear localization signal (NLS) at the C-terminus.

QKI is a member of the signal transduction and activation of RNA (STAR) family, which belongs to the hnRNP K-homology domain protein family (Kondo et al., 1999; Galarneau and Richard, 2005). QKI is located on human chromosome 6 and mouse chromosome 17 and contains an RNA-binding motif in KH domain, which is flanked by two QUA domains (Qua1 and QUA2) (Figure 1). The QUA domain plays important roles in the formation of homo- or heterodimers and is also required for sequence-specific RNA binding (Chen and Richard, 1998; Chenard and Richard, 2008; Beuck et al., 2012; Teplova et al., 2013). In addition to these functional domains, a tyrosine cluster is located within the proline-rich PXXP motif, which can be the critical site to be phosphorylated by Src kinases, giving an additional intracellular signaling pathway to regulate QKI function (Zhang et al., 2003). QKI specifically binds RNA via the Quaking Response Element (QRE) (Galarneau and Richard, 2005). QKI has three major alternatively spliced isoforms, QKI-5, QKI-6, and QKI-7. These isoforms contain identical sequences from exons 1 to 6, but differ in exons 7 and 8 that contribute to QKI carboxy-terminal end (Figure 1; Hardy et al., 1996; Teplova et al., 2013), suggesting that QKI carboxy-terminal is important to various RNA-processing functions. QKI-5 exhibits a nuclear localization signal (Wu et al., 1999; Pilotte et al., 2001) and has been shown to play a major function in pre-mRNA splicing regulation (Wu et al., 2002; Zong et al., 2014; Darbelli et al., 2016; de Bruin et al., 2016a; Li et al., 2018; Caines et al., 2019). QKI-6 and QKI-7 lack nuclear localization signals and apparently have different biological functions (Hardy et al., 1996; Pilotte et al., 2001; Larocque et al., 2009; Shi et al., 2019), and they play more important roles in regulating mRNA other posttranscriptional mRNA processing, such as RNA stability and transportation (Doukhanine et al., 2010; Guo et al., 2011; Yamagishi et al., 2016; Hojo et al., 2020).

## QKI Is Involved in Multiple Physiological Systems

QKI is largely ubiquitously expressed but enriched in the heart and central nervous system during embryonic development (Chen et al., 2021). Previously, QKI is known for its important disease-causing contribution to human diseases, especially the neurological disorders, such as schizophrenia (Aberg et al., 2006), 6q terminal deletion syndrome (Backx et al., 2010), myelin disorders (Bockbrader and Feng, 2008), and Alzheimer’s disease (AD) (Farnsworth et al., 2016). Qk^v^ mouse line is a well-known spontaneous mutant mouse line, in which a 1 Mb promoter/enhancer region is deleted at the upstream of the *Qki* transcription start site and results in myelination defect in the central nervous system (Sidman et al., 1964; Chenard and Richard, 2008), thus confirming the relevance of its role in causing human neurological diseases. Recently, QKI-5 was found significantly downregulated in the peripheral blood of patients with neuromyelitis-optica (NMO) and multiple sclerosis (MS). Both of the disease conditions are largely due to enhanced inflammation, which mediates the alteration in demyelination of the central nervous system (CNS) (Lavon et al., 2019), further supporting the critical contribution of QKI to neuronal disorders.

Over the past several years, these *Qki* mutant mouse models and the use of pluripotent human stem cell differentiation system has revealed a series of important functions of QKI in much broader biological systems, such as cardiovascular development and function, monocyte to macrophage differentiation (de Bruin et al., 2016a), bone metabolism (Jacome-Galarza et al., 2019; Rauwel et al., 2020), and cancer pathogenesis (Darbelli and Richard, 2016). In this mini review, we will focus on the emerging roles of QKI in regulating cardiovascular development and function and its potential link to cardiovascular pathophysiology.

## QKI Safeguards Myofibrillogenesis in Cardiac Development and Function

The heart is the first organ to function during embryonic development (Brand, 2003). Qki expression is found in the developing mouse heart as early as E7.5 and maintained at a high level in cardiomyocytes in all four chambers of adult hearts (Kondo et al., 1999; Noveroske et al., 2002; Chen et al., 2021). In addition, the temporal expression pattern of QKI is analyzed in a human embryonic stem cell (hESC)-to-cardiomyocyte differentiation system. QKI-5 is the main isoform in hESCs, cardiogenic progenitor cells, and differentiated cardiomyocytes and is significantly elevated at the stage that cardiogenic progenitors transition to early differentiated cardiomyocytes, indicating that QKI function is likely more relevant in the early cardiogenic process (Chen et al., 2021). QKI-6 and QKI-7 are maintained at much lower expression levels (Chen et al., 2021), suggesting that QKI-6 and QKI-7 are not essential to the cardiogenic process (Chen et al., 2021).

The hESC-to-cardiomyocyte differentiation process provides a unique *in vitro* platform for testing cardiomyocyte differentiation and genetic pathways that contribute to heart development, maturation and function. QKI-deficient mutant hESC lines (hESCs-*QKI*^*del*^) are capable of differentiating into monolayer cardiomyocyte sheets but have dramatically weaker and asynchronous spontaneous-beating activity. Transcriptomic analysis at a single-cell resolution confirms that hESC-*QKI*^*del*^ largely maintains pluripotency, self-renewal activity, and early cardioprogenitor differentiation ability but has a significant defect in becoming functional cardiomyocytes. Genes involved in contractile function, such as *ACTN2* (α-actinin), *MYL2* (myosin light chain 2), *MYH7* (β-myosin heavy chain), *ANKRD1* (ankyrin repeat domain 1), *TNNI3* (troponin I3), *ACTA1* (actin alpha cardiac muscle 1), and *TAGLN* (transgelin), are downregulated significantly in cardiomyocytes derived from hESCs-*QKI*^*del*^, suggesting that the altered contractile function in QKI-deficient cardiomyocyte sheet is mainly caused by the altered regulation of sarcomere genes. Interestingly, a replicate multivariate analysis of transcript splicing (rMATS) revealed altered alternative splicing in Z-line proteins, such as ACTN2, NEBL, ABLIM1, PDLIM5, and the proteins connecting the Z-line to the M-line in the sarcomere, such as TITIN. The Z-line provides an essential anchor for sarcomerogenesis and contractile function (Frank et al., 2006). Importantly, altered *ACTN2* splicing creates a premature STOP codon that leads to the activation of non-sense-mediated decay (NMD) and consequently to the striking downregulation of *ACTN2* expression (Chen et al., 2021). Consistent with this finding, immunofluorescence staining shows largely disorganized myofibrils lacking striated structure in QKI-deficient cardiomyocytes (Li et al., 2003; Chen et al., 2021). Furthermore, the analysis of a *Qki*-deficient mouse model confirms the essential role of QKI in cardiac myofibrillogenesis and function (Chen et al., 2021). Consistent with these findings, another recent work compared unique alternative splicing events in three definite cell lineages differentiated from hESCs, namely, the endoderm, cardiogenic mesoderm and neuroectoderm, and showed that QKI plays a critical role in lineage toward cardiac mesoderm and the formation of cardiomyocytes, which likely occurs via its regulation of alternative splicing of Bridging Integrator1/Amphiphysin 2 (BIN1) (Fagg et al., 2020). Of note, BIN1 was previously known for its function in regulating cardiac development (Muller et al., 2003) and myofibrillogenesis (Hong et al., 2014). Collectively, these recent investigations have highlighted an indispensable role of QKI in cardiogenesis.

## QKI Is Involved in Cardiac Pathophysiology and Pathogenesis

A two-stage genome-wide association study identified *QKI* as a potential locus associated with myocardial infarction and coronary heart disease, suggesting a potential role of QKI in heart function and disease (Dubé et al., 2016). A correlation analysis comparing coronary heart susceptibility genes and the circadian rhythm pathway showed that QKI is among the 9 candidate genes involved in crosstalk between circadian rhythm and heart failure (Yan et al., 2018). Furthermore, QKI is identified in association with dilated cardiomyopathy (DCM) and cardiac fibrosis (Chothani et al., 2019). These data suggest that QKI is not only critical for cardiac formation but also likely has an important function in regulating cardiac pathophysiology and heart disease pathogenesis.

Guo et al. showed that QKI can constrain ischemia/reperfusion (H/R)-induced cell death in cardiomyocytes by antagonizing the elevation of the proapoptotic transcription factor FoxO1 in response to cardiac injury (Guo et al., 2011). FoxO1 activates two crucial pathways, nitrosative stress and ER (endoplasmic reticulum) stress, thus leading to H/R injury hypersensitivity and cardiomyocyte death in diabetic cardiomyocytes. QKI-5 can suppress the expression of FoxO1 by reducing FoxO1 mRNA stability in cardiomyocytes, thus acting as a protector against diabetic cardiomyopathy (Guo et al., 2014). QKI may also function as a protective factor in doxorubicin (DOX)-mediated cardiotoxicity by regulating the circular RNAs in the heart, including Titin, formin homology 2 domain containing 3 (Fhod3), and striatin/calmodulin-binding protein 3 (Strn3) (Gupta et al., 2018). In addition, QKI expression may be regulated by two microRNAs, miR-155 and miR-31-5p. The elevated level of QKI obtained by inhibiting miR-155 has been shown to repress cardiomyocyte apoptosis, thus improving cardiac function (Tili et al., 2015; Guo et al., 2019). Similarly, miR-31-5p promotes DOX-induced myocardial apoptosis by suppressing QKI expression (Ji et al., 2020). Consistently, overexpression of QKI-5 drastically repressed H/R-induced expression of activated caspase-3 and the generation of reactive oxygen species (ROS) that exacerbate cardiomyocyte apoptosis (Wang et al., 2017). This series of studies strongly indicates the important cardioprotective function of QKI in the adult heart. However, the underlying mechanism remains largely elusive and certainly requires further investigation.

## QKI Is Involved in Vascular Smooth Muscle Differentiation

QKI expression can be strongly induced in vascular smooth muscle cells (VSMCs) in response to vascular injury and is shown to be involved in VSMC dedifferentiation via its regulation of alternative splicing of Myocd, a well-known master transcription factor in smooth muscle differentiation (van der Veer et al., 2013). Perturbation of QKI activity alters pathogenetic and fibroproliferative responses to vascular injuries (van der Veer et al., 2013). Another interesting study suggests that miR-214 directly targets 3′UTR region of QKI-6/7 mRNA, but not QKI-5 mRNA, and potentially functions as a negative regulator of QKI-6/7 expression during VSMC differentiation (Wu et al., 2017), suggesting an important QKI/isoform-mediated mechanism in regulating VSMC differentiation and function.

A recent study demonstrated that QKI-6 regulates HDAC7 splicing and promotes VSMC differentiation from induced pluripotent stem cells (iPSCs). Remarkably, QKI-6-overexpressing smooth muscle cells show a greater contractile ability, and when cocultured with QKI-5-overexpressing endothelial cells, QKI-6-overexpressing smooth muscle cells exhibit a greater angiogenic potential (Caines et al., 2019), suggesting a unique corporative yet isoform-specific function between vascular endothelial and smooth muscle physiology.

## QKI Is Involved in Vascular Endothelial Cell Differentiation and Function

QKI is highly expressed in both macro- and microvascular endothelial cells (de Bruin et al., 2016b). Several lines of study suggest that QKI is involved in endothelial differentiation and function. In the iPSC to endothelial cell differentiation system, QKI-5 (but not QKI-6 and QKI-7) is induced (Cochrane et al., 2017). QKI-5 directly binds to the 3′UTR of STAT3 to stabilize the expression of STAT3 and thus enhances VEGF receptor 2 (VEGFR2)-mediated signaling and VEGF secretion, which promotes the differentiation of endothelial cells and vasculogenesis (Cochrane et al., 2017). Apparently, QKI-5 can stabilize CD144 and CD31 during endothelial differentiation. Overexpressing QKI-5 in iPSC-derived endothelial cells can greatly improve angiogenesis and neovascularization as well as blood flow recovery in experimental hind limb ischemia, demonstrating the critical role of QKI in endothelial cell differentiation and function (Cochrane et al., 2017).

Interestingly, QKI-7 can be significant upregulated in mouse iPSC-derived endothelial cell when exposed to hyperglycemia mimicking diabetic pathophysiological condition (Yang et al., 2020). Importantly, QKI-7 is also upregulated in human iPSC-derived endothelial cells from diabetic patients and human coronary arterial endothelial cells isolated from diabetic donors (Yang et al., 2020). Under diabetic conditions, QKI-7 directly binds and promotes the degradation of the mRNAs of CD144, NLGN1, and TSG6, thus leading to vascular endothelial cell dysfunction (Yang et al., 2020). CD144 is recognized as an endothelial adhesion molecule that is involved in maintaining barrier integrity and promoting angiogenesis. TSG6 and NLGN1 have been shown to regulate endothelial cell–matrix interactions and play an important role in vasculogenesis. Knockdown of QKI-7 in diabetic mice restores endothelial function and promotes blood flow recovery in the ischemic hindlimb (Yang et al., 2020). In addition, laminar shear stress has been shown to induce QKI expression in endothelial cells by the transcription factor KLF2 (de Bruin et al., 2016b). Knockdown of QKI perturbs the endothelial barrier function *in vitro*, which likely occurs via its activity in binding to the 3′UTR of VE-cadherin and β-catenin mRNA and the regulation of VE-cadherin and β-catenin mRNA translation (de Bruin et al., 2016b). Collectively, these data suggest that QKI-5 and QKI-6 provide positive regulation to angiogenesis via endothelial cells and VSMCs, respectively, while QKI-7 negatively regulates endothelial function, indicating the key functional influences of the unique C-terminal sequences on the isoform-specific functions.

## Perspectives

**FIGURE 2:**
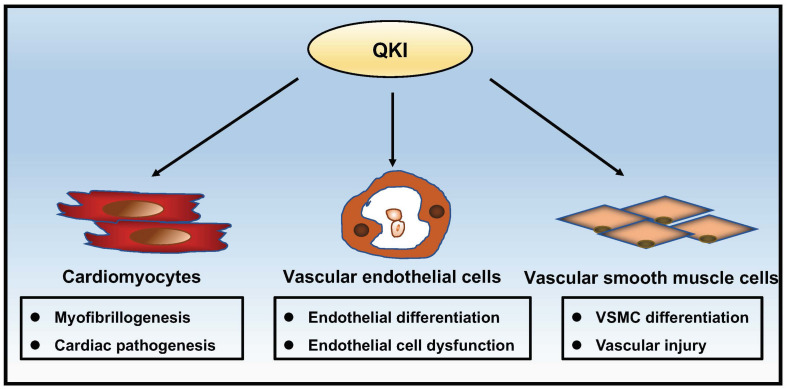
Schematic summary of the complex roles of QKI in cardiac and vascular development and function.

This mini review summarizes a series of recent exciting data on the physiological role of QKI in regulating numerous important features of cardiovascular development and function. As shown in Figure 2, these emerging findings not only provide a critical understanding of QKI as an important regulator in pre-mRNA processing but also reveal its potential contribution to the pathogenesis of cardiovascular diseases and potential use as a therapeutic target in treating these diseases. Recently, QKI has been identified as one of the top 25 genes associated with aging (Arabfard et al., 2019). Interestingly, an earlier genome-wide association study of a cohort with 263 cognitively intact Amish individuals at age 80 or older linked aging to 6q25-27, a chromosomal region containing the QKI gene (Edwards et al., 2013). Although this genetic association will need further confirmation, the close association of QKI in cardiovascular diseases and the unique isoform-specific function of QKI among different cell types that shape the cardiovascular system have been recognized. The combined use of the hESC/hiPSC experimental system and mouse models will facilitate a better understanding of the underlying molecular mechanism by which QKI transcription and isoform switching are regulated during normal developmental processes and disease progression in the future.

## Author Contributions

XC and JY drafted the manuscript. DC and DX revised the manuscript. ZZ, YL, and WS finalized the manuscript. All authors contributed to the article and approved the submitted version.

## Conflict of Interest

The authors declare that the research was conducted in the absence of any commercial or financial relationships that could be construed as a potential conflict of interest.
